# Timing of Antioxidant Gene Therapy: Implications for Treating Dry AMD

**DOI:** 10.1167/iovs.16-21272

**Published:** 2017-02

**Authors:** Manas R. Biswal, Pingyang Han, Ping Zhu, Zhaoyang Wang, Hong Li, Cristhian J. Ildefonso, Alfred S. Lewin

**Affiliations:** 1 Department of Molecular Genetics and Microbiology, University of Florida College of Medicine, Gainesville, Florida, United States; 2 Department of Ophthalmology, University of Florida College of Medicine, Gainesville, Florida, United States; 3 Department of Ophthalmology, Shanghai Ninth People's Hospital, Shanghai Jiaotong University School of Medicine, Huangpu District, Shanghai, China

**Keywords:** gene therapy, age-related macular degeneration, superoxide dismutase 2, retinal pigment epithelium, geographic atrophy

## Abstract

**Purpose:**

To investigate whether antioxidant gene therapy protects the structure and function of retina in a murine model of RPE atrophy, and to determine whether antioxidant gene therapy can prevent degeneration once it has begun.

**Methods:**

We induced mitochondrial oxidative stress in RPE by conditional deletion of *Sod2*, the gene for manganese superoxide dismutase (MnSOD). These mice exhibited localized atrophy of the RPE and overlying photoreceptors. We restored *Sod2* to the RPE of one eye using adeno-associated virus (AAV) by subretinal injection at an early (6 weeks) and a late stage (6 months), injecting the other eye with an AAV vector expressing green fluorescent protein (GFP). Retinal degeneration was monitored over a period of 9 months by electroretinography (ERG) and spectral-domain optical coherence tomography (SD-OCT). Immunohistochemical and histologic analyses were conducted to measure oxidative stress markers and to visualize retinal structure.

**Results:**

One month after delivery, the AAV-*Sod2* injection resulted in production of MnSod in the RPE and negligible expression in the neural retina. Electroretinography and OCT suggested no adverse effects due to increased expression of MnSOD or subretinal injection. Decrease in the ERG response and thinning retinal thickness was significantly delayed in eyes with early treatment with the *Sod2* vector, but treatment at 6 months of age did not affect the ERG decline seen in these mice.

**Conclusions:**

We conclude that antioxidant gene therapy may be effective in preventing the detrimental effects of oxidative stress, but may not be beneficial once substantial tissue damage has occurred.

Age-related macular degeneration (AMD) is one of the leading causes of irreversible visual dysfunction among the elderly.^1^ Visual dysfunction in AMD patients is characterized as either “dry” AMD or “wet” or neovascular AMD or both. The advanced form of dry AMD, also termed geographic atrophy (GA), is caused by death of macular retinal pigment epithelial (RPE) cells and degeneration of photoreceptors. In neovascular AMD, choroidal blood vessels invade the retina, causing vascular leakage and scarring within retina. In either of the forms, central vision is severely affected, leading to loss of independence. Even though treatments are available for wet AMD, there is no effective treatment for GA, though research on replacement of the RPE is in progress.^2,3^

Damage to the RPE and photoreceptors has been linked to progressive vision loss in the dry form of AMD. Animal models and the Age-Related Eye Disease Study (AREDS) trial strongly support a role for oxidative stress in the development of AMD.^4,5^ Because of the importance of oxidative stress as a contributing factor in AMD, treatments that reduce the accumulation of reactive oxygen species (ROS) may be valuable as therapeutics, though antioxidant therapy has shown more benefit in preventing progression to wet AMD than to GA.^6–9^ Our lab has developed a mouse model of GA in which the knockdown or deletion of *Sod2*, the gene for manganese superoxide dismutase (MnSOD), in the RPE leads to elevated oxidative stress, causing some of the features of GA including damage to the RPE and Bruch's membrane and death of photoreceptors.^10,11^ Depletion of MnSOD in the RPE in mice shows accumulation of lipofuscin-like fluorescent aggregates containing A2E and Iso-A2E.^12^ While markers of oxidative stress are elevated by 2 months of age in this model, reductions in ERG a-wave and b-wave amplitudes and thinning of the outer nuclear layer (ONL) are not statistically significant until 6 months of age. Therefore, we reasoned that reducing oxidative stress in this interval (between 2 and 6 months) had the prospect of preventing retinal degeneration in these mice. We have also used this model for testing drug therapy for GA^13–15^ starting at weaning and have shown that treatment with antioxidants reduces free radical accumulation in the RPE and outer nuclear layer.^16^

Because gene therapy can be targeted to the retina and RPE^17–20^ and potentially provide long-term protection against oxidative stress, this approach may be advantageous in treating a chronic condition like advanced dry AMD. Timing of therapy is a critical issue, however. Oxidative stress may contribute to the pathologic changes of RPE and retina, but will antioxidant therapy be beneficial in preventing GA after clinical signs have appeared? In this study we inquired whether adeno-associated virus (AAV)–mediated gene delivery of *Sod2* could reverse the impact of RPE-specific deletion of the same gene at two time points, early and late after deletion of the gene. We found that early replacement of *Sod2* in RPE delayed retinal degeneration, whereas replacement of *Sod2* after substantial RPE damage had already occurred did not prove effective, suggesting that antioxidant gene therapy may be useful as a preventive but not as a therapy for GA.

## Methods

### Study Design

**Figure 1:**
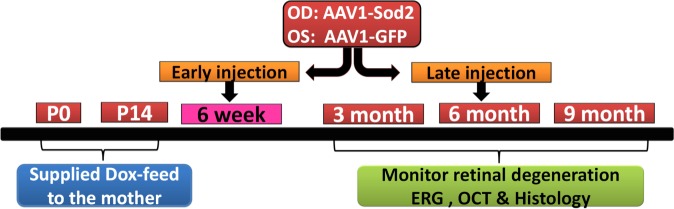
Study design. Two age groups of mice were used to design the experiment, one group of mice at 6 weeks of age (early stage) and another group of mice at 6 months of age (late stage). The AAV1-*Sod2*-*myc* experimental vector was injected in one eye, and the contralateral eye was injected with control vector AAV1-*GFP*. The mice were examined by ERG and SD-OCT at 3, 6, and 9 months for early gene delivery and at 9 months of age for late gene delivery. After 9 months, changes in retinal ultrastructure in response to *Sod2* delivery were examined using electron microscopy. *Sod2* expression was determined by Western blotting and its effect in reducing markers of oxidative stress was quantified by ELISA.

Two age groups of mice were used to design the experiment, one group of mice at 6 weeks of age (early stage), and another group of mice at 6 months of age (late stage) (Fig. 1). The AAV1-*Sod2* experimental vector was injected in one eye, and the contralateral eye was injected with control vector AAV1–green fluorescent protein (GFP). The mice were analyzed up to 9 months of age following early gene delivery and to the same age following late gene delivery as described in the figure legend.

### Animal Model

This study adhered to the ARVO Statement for the Use of Animals in Ophthalmic and Vision Research, and all animal procedures were approved by the University of Florida Institutional Animal Care and Use Committee. The mice used for the experiment were transgenic for P_VMD2_-rtTA and tetO-P_hCMV_cre and were homozygous for *Sod2* containing loxP sites surrounding exon 3.^10,21^ This model and the procedure for induction of cre recombinase has been described by Mao et al.^10^ For procedures such as subretinal injection, electoretinography (ERG), fundus imaging, and spectral-domain optical coherence tomography (SD-OCT), mice were anesthetized by intraperitoneal injection of ketamine (95 mg/kg) and xylazine (8 mg/kg). Procedures for local anesthesia and dilation were described in detail by Biswal et al.^14^ At the end of experiments, carbon dioxide (>90%) inhalation or intraperitoneal Euthasol (sodium pentobarbital, 150 mg/kg; Virbac, Inc. Fort Worth, TX, USA) injection was used to euthanize the mice.

### AAV Vector

Self-complementary AAV vector was used to clone mouse *Sod2* cDNA, a single copy of myc epitope inserted replacing stop codon of *Sod2* (Supplementary Fig. S1A). A self-complementary AAV vector carrying *GFP* cDNA was used as a control to compare the effects of AAV injection. Expression of both genes was driven by a truncated version of the chimeric chicken β-actin proximal promoter and the immediate early enhancer of cytomegalovirus (the CBA promoter).^22^ Adeno-associated virus 1 was purified by the Vector Core of the Center for Vision Research at the University of Florida using Iodixanol gradients and anion exchange chromatography.^23^ The stock concentration of purified AAV1-*Sod2* and AAV1-GFP was 1 × 10^12^ genome copies (gc) per milliliter.

### Subretinal Injection

Adeno-associated virus (10^9^ viral particles in 1 μL) was injected subretinally via the pars plana as described by Timmers et al.^24^ We examined all animals by SD-OCT 2 weeks after subretinal injection, and all animals with unhealed retinal detachments were excluded from further analysis.

### Western Blot

Protein lysates from RPE/choroid were placed in sample buffer containing dithiothreitol and boiled for 6 minutes at 95°C. Equal amounts of protein were separated using SDS polyacrylamide gel electrophoresis and transferred into a polyvinylidene difluoride (PVDF) membrane using the iBlot system (Invitrogen, Thermo Fisher Scientific, Waltham, MA, USA). This membrane was blocked with a blocking buffer from Li-Cor Biosciences (Lincoln, NE, USA) for 1 hour at room temperature and incubated overnight with the designated primary antibody at 4°C. To detect MnSOD we used a primary rabbit polyclonal antibody from Abcam (Cat. no. ab13533, 1:1000 dilution; Cambridge, MA, USA) and a donkey anti-rabbit secondary antibody from Li-Cor Biosciences (Cat. no. 926-32213, 1:5000 dilution); to detect expression of the exogenously delivered *Sod2*-*myc* gene, we used a mouse anti-myc antibody from Invitrogen (Cat. no. 04-1117, 1:5000 dilution), and the secondary antibody was donkey anti-mouse from Li-Cor Biosciences (Cat. no. 926-68072, 1:5000 dilution). Rabbit anti-alpha–tubulin primary antibody from Abcam (Cat. no. ab7291, 1:10,000 dilution) and a donkey anti-rabbit secondary antibody from Li-Cor Biosciences (Cat. no. 926-32213, 1:5000 ratio) were used to detect tubulin.

### Electroretinography

Scotopic ERG recordings were made as described in our previous paper.^14^ Briefly, we recorded scotopic a- and b-wave ERG responses at three different light intensities (−20 dB [0.02 cds/m^2^], −10 dB [0.18 cds/m^2^], 0 dB [2.68 cds/m^2^]) from both eyes using an LKC UTAS Visual Electrodiagnostic System with a BigShot full-field dome (LKC, Gaithersburg, MD, USA). To examine RPE responses, c-wave amplitudes from each eye were measured using Espion full-field ERG system at a flash intensity of 20 dB (50 cds/m^2^), and the results were compared between treated and untreated eyes at one time point.

### Spectral-Domain Optical Coherence Tomography

High-resolution SD-OCT images were obtained by employing Envisu SD-OCT ophthalmic imaging system (Bioptigen, Durham, NC, USA) as described in our previous paper.^14^ The outer nuclear layer (ONL) was measured at four different locations (temporal, nasal, superior, and inferior) at 0.35-mm distance from the optic nerve head (ONH). Upon averaging ONL thickness from each eye, the results were compared between treated and untreated eyes. We also used Bioptigen's autosegmentation program to measure layers of the retina and RPE.

### Evaluation of Oxidative Stress Markers 8-OHdG and Nitrotyrosine

Immunohistochemistry for 8-hydroxy-deoxyguanosine (8-OHdG) was conducted according to the previously published protocol.^14^ The nitrotyrosine (3-nitrotyrosine, 3NT) competitive enzyme-linked immunosorbent assay (ELISA) (Abcam, Cat. no. ab113848) was used to estimate the nitrotyrosine-modified protein levels from the retina/RPE/choroid sample, performed according to the manufacturer's protocol. Briefly, 96-well microplates were coated with nitrotyrosine containing antigen overnight. The eye cup containing retina/RPE/choroid was dissected separately from the eyes treated with either AAV1-*Sod2* or AAV1-*GFP* vector 45 days following subretinal injection. The samples were collected in 150 μL phosphate-buffered saline (PBS) and disrupted by sonication for 10 seconds on ice. The samples were further diluted for ELISA. Triplicates of each treatment and triplicates of each test standard were used in the assay. Being the competitive assay, the increased 3-NT in the sample results in a reduced colorimetric reaction at 600 nm.

### Light and Electron Microscopy

For light and electron microscopy, mice were injected with an overdose of sodium pentobarbital and perfused with a mixture of PBS containing 2% paraformaldehyde and 2.5% glutaraldehyde. The fixed eyes were collected and processed according to the procedure described in our previous paper.^14^

### Statistical Analysis

The statistical software GraphPad Prism (version 5.0; Graph Pad Software, Inc., San Diego, CA, USA) was used to analyze the data. All reported *P* values were calculated using the 2-tailed Mann-Whitney test as indicated in the legends, and a *P* value of <0.05 was considered significant. All data are represented as mean ± SEM unless otherwise indicated.

## Results

### RPE-Specific *Sod2* Deletion and Overexpression

**Figure 2:**
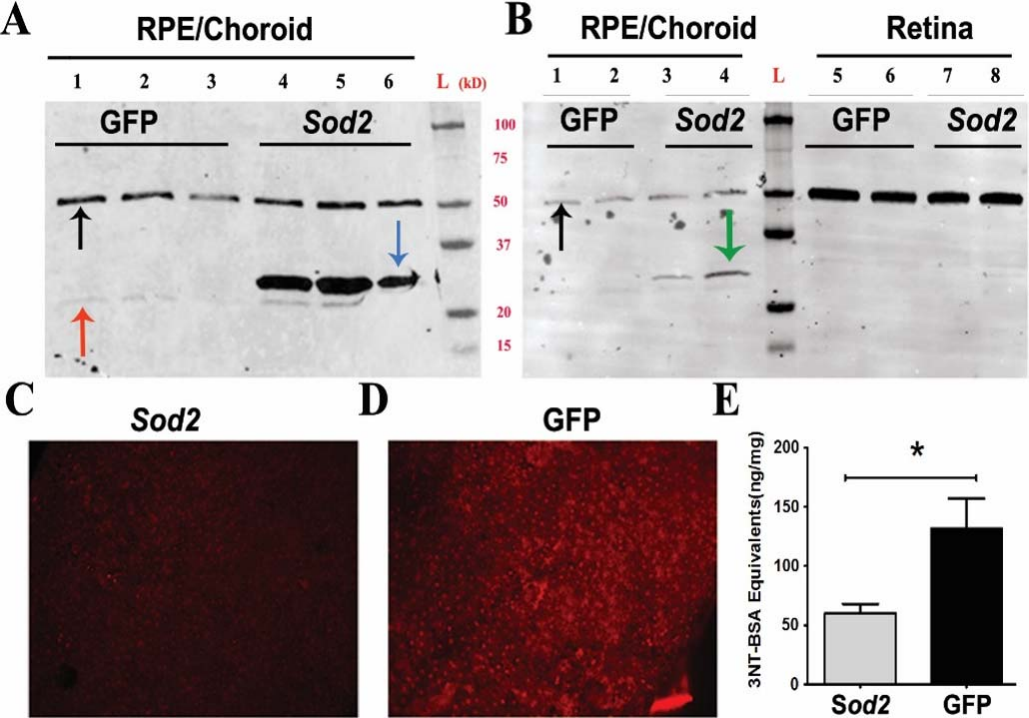
RPE-specific *Sod2* expression and its effect on oxidative stress. For Western blot analysis, the mice were injected at 6 weeks and tissue was harvested at 2.5 months of age. Compared to eyes injected with the control AAV1-*GFP* vector (*lanes 1*, *2*, *3*, *red arrow*), *Sod2* expression was significantly increased (**A**) in the RPE/choroid of *Sod2*^flox/flox^/*VMD2*-*cre* mice (*lanes 4*, *5*, *6*, *blue arrow*) injected with the AAV1-*Sod2* vector using a MnSOD-specific antibody. The *lower faint band labels* for the endogenous *Sod2* and slower-migrating dense bands are exogenous MnSOD-myc. Using a myc antibody, myc-tagged *Sod2* expression was detected (**B**) only in RPE/choroid in vector-injected eyes (*green arrow*, *lanes 3*, *4*), but myc-tagged *Sod2* was not seen in RPE from AAV-*GFP*–injected eyes. The *black arrow* indicates the α-tubulin loading control. For RPE flat-mount analysis, the mice were injected at 6 weeks, and tissue was harvested at 3 months. Fluorescence images of flat-mounted RPE derived from treated (**C**) (AAV1-*Sod2*)–injected and control-treated (AAV1-*GFP*) (**D**) eyes stained with primary antibody to 8-OH-deoxyguanosine, a marker of oxidative damage to DNA. In response to AAV1-*Sod2* therapy, eyes showed a reduced level of 3-nitrotyrosine (**E**) compared to control eyes treated with AAV-*GFP* (*n* = 5) by ELISA. *P = 0.014.

Our mouse model (*Sod2*^flox/flox^/*P_VMD2_*-*rtTA; tetO*-*P*_hCMV_*cre*) involves RPE-specific deletion of *Sod2*, using the cre-lox system, and results in a gradual retinal degeneration demonstrated by reduction in electroretinogram (ERG) amplitudes and in the thickness of the ONL that are statistically significant by 6 months of age.^10^ In this study, we examined whether *Sod2* gene therapy could restore visual function and retinal degeneration in the *Sod2*^flox/flox^/*VMD2*-*cre* mice. Serotype 1 AAV selectively transduces RPE cells following subretinal injection in mice.^25^ To enhance the expression of MnSOD in the RPE, we injected an AAV1 vector containing myc epitope-tagged mouse *Sod2* cDNA into the subretinal space of one eye of 6-week-old or 6-month-old *Sod2*^flox/flox^*/VMD2*-*cre* mice (Supplementary Fig. S1A). To study the impact of injection or the delivery of AAV particles, we treated the contralateral eyes from the same animals with an AAV1 vector expressing humanized GFP. In control eyes, GFP expression was observed over 60% to 80% of the retina by fluorescence fundus imaging, suggesting efficient delivery of vector (Supplementary Fig. S1B). We found that MnSOD protein levels were reduced by 50% to 60% in eyes of doxycycline-induced *Sod2*^flox/flox^/*VMD2*-*cre* mice compared to *Sod2*^flox/flox^/*VMD2*-*cre* mice not induced with doxycycline (data not shown). To quantify exogenous *Sod2* protein levels, the level of AAV-delivered MnSOD expression was examined 1 month following injection (Fig. 2A) using a MnSOD-specific antibody. To validate the RPE selectivity of the vector, retina and RPE/choroid tissue samples were collected separately from a cohort of mice at 1-month post injection. Using a myc antibody, myc-tagged MnSOD was detected in RPE/choroid samples treated with AAV1-*Sod2* vector (Fig. 2B). As expected from the tropism of AAV1, negligible expression was observed in the neural retina.

### Reduction in Markers of Oxidative Stress

By immunohistochemistry and ELISA, we have previously shown that *Sod2* deletion in RPE increases markers of oxidative stress.^13,14^ Upon staining RPE flat mounts with antibody to 8-OHdG, we found a significant reduction in labeling of 8-OHdG fluorescence in treated eyes at the age of 3 months (Fig. 2C) compared to control eyes (Fig. 2D). Deletion of MnSOD increases the level of superoxide that reacts with nitric oxide to produce peroxynitrite (ONOO−), and adducts are formed by reaction of peroxynitrate with tyrosine residues. To determine if *Sod2* vector treatment reduced the burden of modified proteins in these mice, we collected the eyes 1 month after injection of vector (injected at 6 weeks, tissue harvested at 2.5 months). The protein samples from the vector-treated eyes were assayed for 3NT by ELISA at the age of 3 months. We found a 54% reduction in levels of nitrotyrosine in treated eyes compared to control eyes (Fig. 2E). We expect that injection of AAV-*Sod2* reduced oxidative stress in the mouse retina based on previous results,^26,27^ but we did not specifically measure the 8-OHdG and peroxynitrate in mice injected at 6 months of age.

### Early Delivery of AAV1-*Sod2* Restores Retinal Function

**Figure 3:**
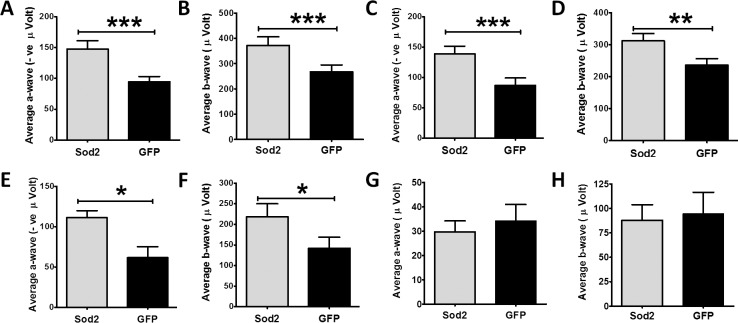
Rescue of retinal function detected by electroretinography. (**A**–**F**) represent early delivery of vector, whereas (**G**, **H**) represent late delivery of vectors. RPE-specific delivery of *Sod2* at 6 weeks of age preserved a-wave amplitudes at 3 months ([**A**] *n* = 18, ***P = 0.007), 6 months ([**C**] *n* = 18, ***P = 0.007), and 9 months ([**E**] *n* = 7, *P = 0.023) following flashes at a light intensity of 2.68 cds/m^2^ (rod plus cone response). At the same light intensities, we saw preservation of b-wave amplitudes at 3 months ([**B**] *n* = 18, ***P = 0.003), 6 months ([**D**] *n* =18, **P = 0.005), and 9 months ([**F**] *n* = 7, *P* = 0.016) following early treatment. We did not see any improvement in a-wave (**G**) and b-wave (**H**) responses from eyes at 9 months (3 months after treatment).

Three months following treatment with AAV1-*Sod2*, the experimental mice were examined by dark-adapted electroretinography (ERG). In eyes treated with AAV-*Sod2*, a-wave amplitudes decreased only 18% between 3 and 9 months following injection at 6 weeks of age. Electroretinogram signals from the control-treated eyes were lower at each time point relative to eyes to which *Sod2* had been delivered. At the highest light intensity (2.68 cd/m^2^), treated eyes showed 56%, 59%, and 80% improved a-wave ERG responses compared to control eyes at 3, 6, and 9 months following treatment, respectively (Figs. 3A, 3C, 3E; Supplementary Figs. S2A, S2C, S2E, S2G). Similarly, the b-wave amplitudes at this light intensity in AAV1-*Sod2*–treated eyes were 38%, 32%, and 53% higher than the control-treated eyes at these intervals (Figs. 3B, 3D, 3F; Supplementary Figs. S2B, S2D, S2F, S2H).

### Improved RPE Functional Integrity in Early-Treated Eyes

**Figure 4:**
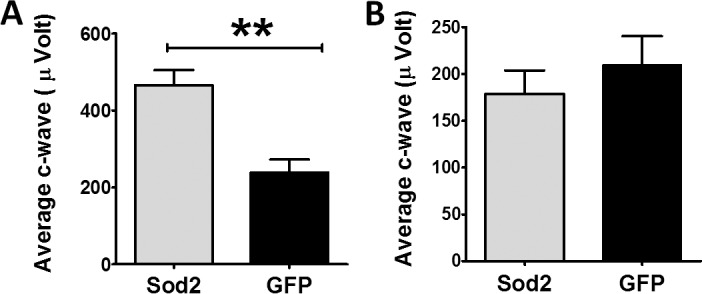
Rescue of c-wave electroretinography. In response to early treatment we observed improved c-wave (RPE response) at 50 cds/m^2^ in early-treated eyes ([**A**] *n* = 10, ***P = 0.005), but we did not see any improvement in c-wave eyes treated with AAV1-*Sod2* at 6 months (**B**).

The c-wave component of the ERG has been correlated with RPE function.^28^ We found that the maximum amplitude of the c-wave was reduced by 39% in doxycycline-induced *Sod2*^flox/flox^/*VMD2*-*cre* mice by 3 months (Supplementary Fig. S3A) compared to uninduced mice. Therefore, we recorded c-wave from the dark-adapted mice 3 months following early treatment. The average c-wave amplitudes were significantly increased (>2-fold) in AAV1-*Sod2*–treated eyes compared to control-treated eyes at this time point (Fig. 4A).

### Structural Rescue by *Sod2* Vector (SD-OCT)

**Figure 5:**
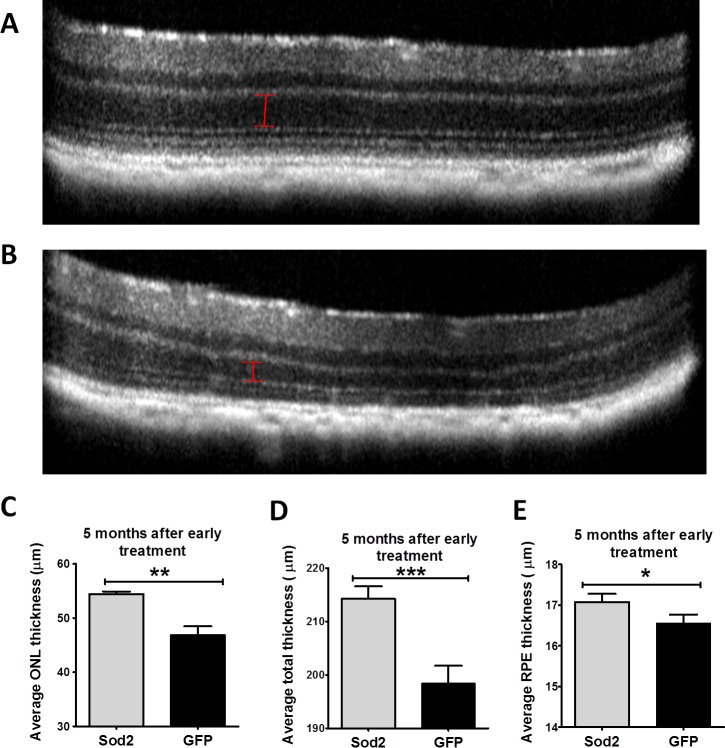
SD-OCT 5 months after early treatment. SD-OCT images (10 individual scans are averaged) of mouse retinas from mice treated early with AAV1-*Sod2* (**A**) compared to control-treated eyes treated with AAV1-*GFP* (**B**). ONL thickness ([**C**] *n* = 7, **P = 0.002), total retinal thickness ([**D**] *n* = 7, ***P = 0.001), and RPE thickness ([**E**] *n* = 7, *P = 0.048) from treated eyes 5 months following early treatment indicated preservation of retinal structures.

**Figure 6:**
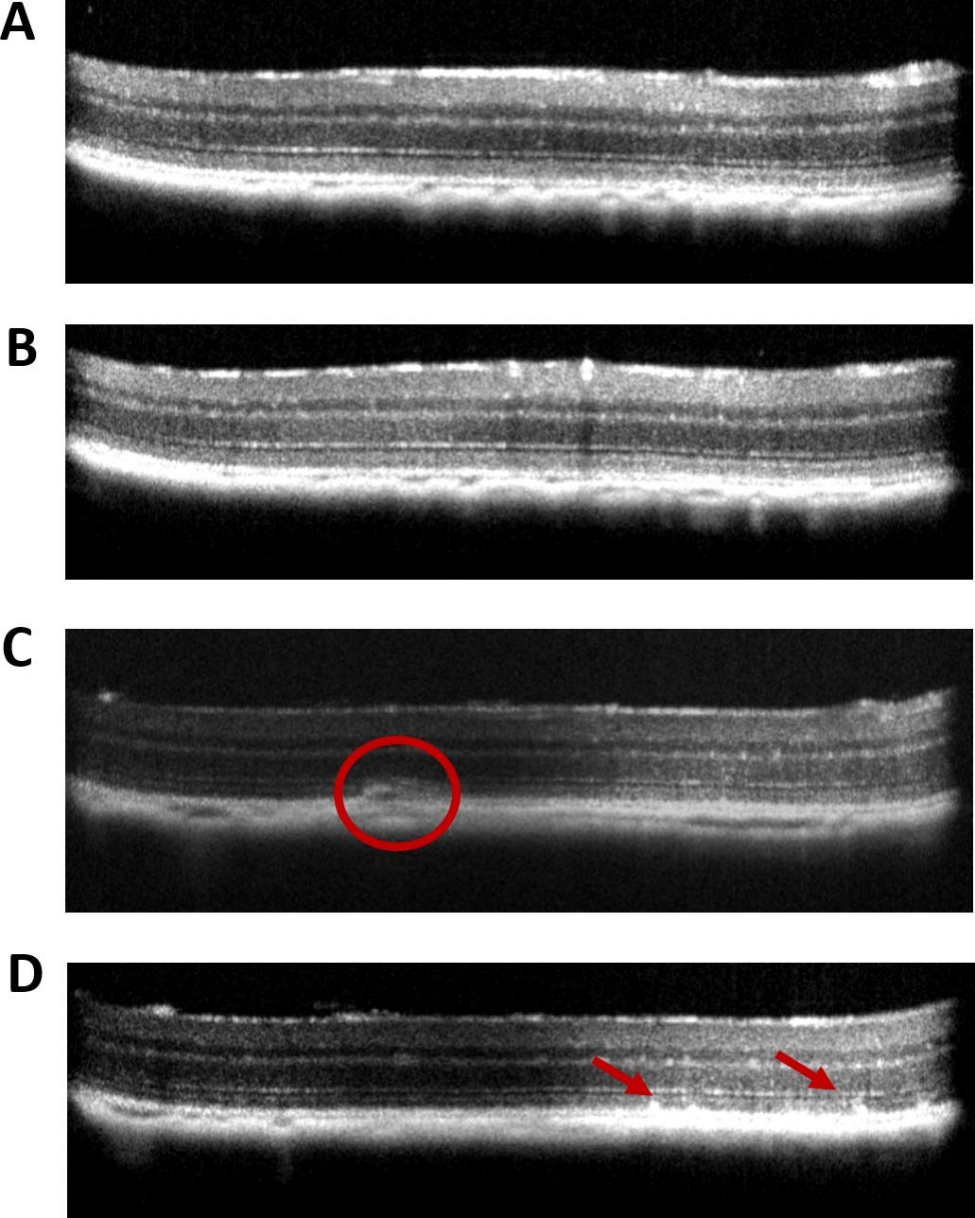
Retina structure by SD-OCT. SD-OCT images of mouse retinas from mice treated early with AAV1-*Sod2* (**A**, **B**) compared to control-treated eyes treated with AAV1-*GFP* (**C**, **D**). Untreated eyes injected with control AAV1-*GFP* vector contained subretinal deposits ([**C**] *red circle*) and reflective spots ([**D**] *red arrows*), but these features were absent in response to early treatment with AAV1-*Sod2* (**A**, **B**).

At 5 and 9 months after early treatment, SD-OCT examinations were carried out to measure the thickness of the retinal layers (Figs. 5A, 5B). Visual inspection of the images revealed thinning of the entire retina and the outer nuclear thickness in control-treated eyes by 5 months post injection with the vectors. To compare structural differences, the ONL was measured at four different locations (temporal, nasal, superior, and inferior) at 0.35-mm distance from the ONH. At 5 months following early treatment, the average ONL thickness was reduced by 13% in control-treated eyes relative to eyes treated with AAV1-*Sod2* (*P* < 0.01) (Fig. 5C). At 9 months, the difference in ONL thickness between treated and control eyes was 18% (*P* < 0.01) (Supplementary Fig. S4A). Upon analyzing the OCT images by the Bioptigen autosegmentation program (Driver), we found preservation of total retinal thickness and RPE thickness in early-treated eyes at 5 months (Figs. 5D, 5E) and 9 months post treatment compared to eyes injected with control vector (Supplementary Figs. S4B, S4C). In control eyes, we observed accumulation of subretinal deposits (circle) and white hyperreflective spots (red arrows) (Figs. 6C, 6D), which were not seen in AAV-*Sod2*–treated eyes (Figs. 6A, 6B).

### Lack of Protection Following Late Treatment

Before late injection with AAV1-*Sod2* we found significant reductions in ERG amplitudes (a-, b-, and c-waves) in our experimental mice (*Sod2*^flox/flox^/*VMD2*-*cre*) compared to *Sod2*^flox/flox^ without the inducible cre transgene (51% for a-wave; 47 % for b-wave, and 52% for c-wave) (Supplementary Figs. S3B, S3C, S3D). In contrast to treatment at 6 weeks of age, subretinal injection of AAV1-*Sod2* at 6 months of age offered no evidence of retinal protection. Three months following late treatment, ERG a-wave and b-wave amplitudes were not significantly different between eyes treated with the experimental or the control vectors (Figs. 3G, 3H). Late treatment with *Sod2* did not show any significant change in c-wave ERG responses (Fig. 4B) between AAV1-*Sod2*–treated and control-injected eyes. Similarly, we did not find a difference in ONL thickness (Supplementary Fig. S5C), total retinal thickness (Supplementary Fig. S5D), or RPE thickness (Supplementary Fig. S5E) in late-treated eyes 3 months post treatment.

### *Sod2* Vector Preserved the Structure of the RPE and Photoreceptors

**Figure 7:**
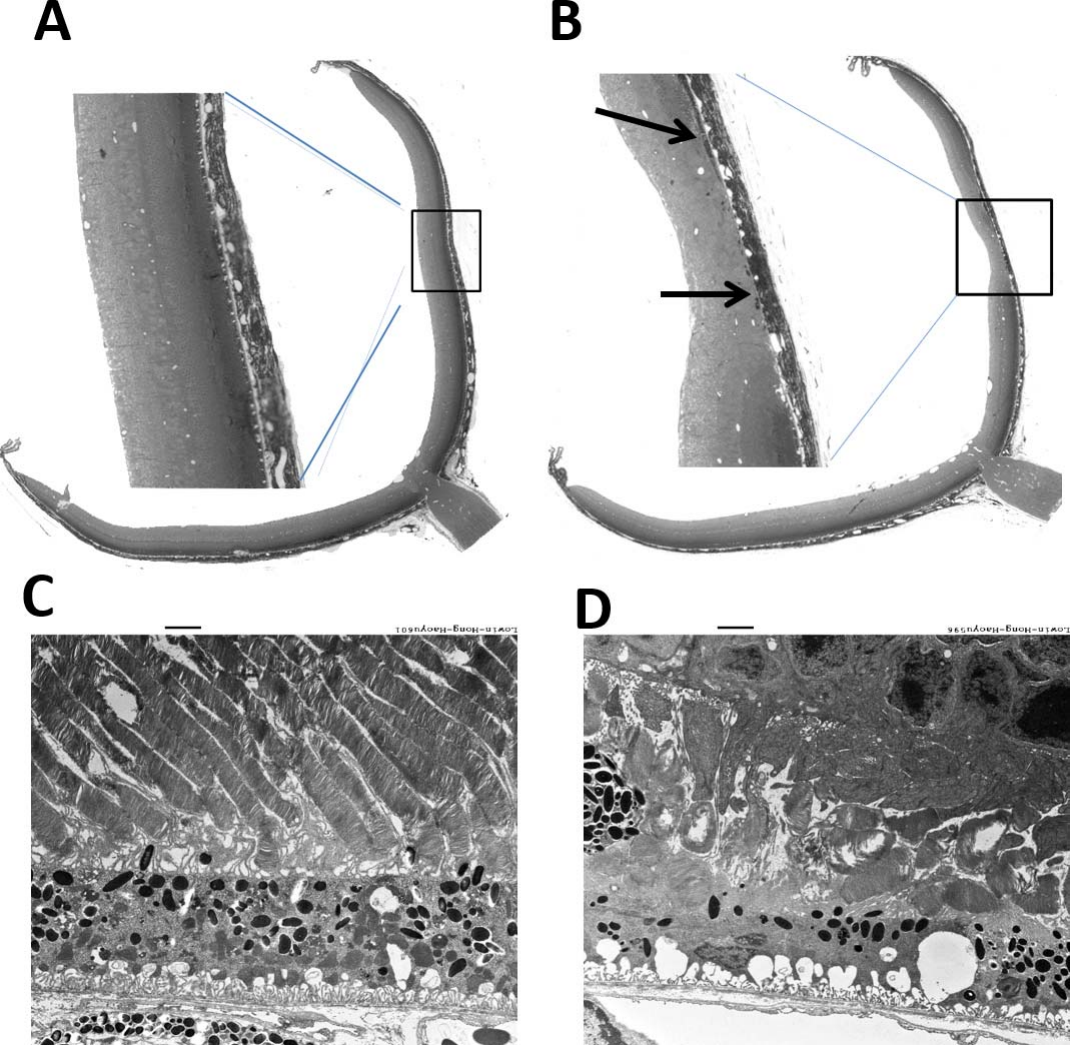
Retina ultrastructure. A light micrograph showed improved retinal thickness in an AAV1-*Sod2*–treated eye (**A**) compared to a control eye (**B**) injected with AAV1-*GFP* vector. Ultrastructure analysis showed preservation of photoreceptors and RPE in an early-treated eye (**C**), whereas the contralateral eye revealed damaged photoreceptors and RPE (**D**). *Scale bars:* 2 μm.

Since we found that early treatment preserved visual function and retinal structure, we processed eyes of mice treated at 6 weeks for microscopic analysis. At 9 months of age, in lower-magnification light micrographs (Figs. 7A, 7B), RPE vacuolization was apparent in both treated and control-treated retinas, but RPE atrophy was a feature only of the control eyes. In electron micrographs, areas of atrophy exhibited thinned RPE and highly vacuolized RPE with disorganized infoldings at the basal surface of RPE cells. Few, if any, mitochondria were detected. Outer segments of photoreceptors were very short, and only one or two layers of nuclei were present in the ONL (Fig. 7D). In the AAV1-*Sod2*–treated eyes (Fig. 7C), rod outer segments were full length but contained cystic spaces and some regions of apparent distension. The RPE layer was much thicker than in control eyes and contained mitochondria normally displaced at the basal surface of cells, and the basal infoldings were more compact than in the control eyes.

## Discussion

Mitochondrial oxidative stress has been implicated in tissue damage, and in the retina mitochondrial dysfunction is reported in AMD eyes.^29–31^ Oxidative damage to the RPE can impact photoreceptors.^15^ Induction of ROS in the absence of sufficient antioxidant system can stimulate the complement cascade and other inflammatory pathways.^6,32,33^ Retinal pigment epithelial cells derived from AMD patients have decreased antioxidative defenses.^34^ Normal aging or cigarette smoking can cause generation of ROS in the retina.^35^

Modeling dry AMD in mice is difficult, since these nocturnal rodents do not rely on cones for central vision and have no anatomic structures analogous to the macula and fovea. Several gene knockout models have been developed that lead to RPE failure and atrophy due to oxidative stress. Mice deficient in Cu, Zn-superoxide dismutase (SOD1) showed oxidative damage and reduced barrier integrity of the RPE.^4^ Nuclear factor (erythroid-derived 2)-like 2 (NRF2)-deficient mice show age-related clinical phenotypes of RPE degeneration and increased presence of autophagic vacuoles in addition to spontaneous choroidal neovascularization.^36^ Mice carrying deletions in hephaestin and ceruloplasmin are also subject to RPE oxidative stress caused by iron overload, and these mice also show RPE atrophy leading to loss of photoreceptors.^37^ Other attempts to model AMD using mice recapitulate other aspects of pathology. For example, mice knocked-in for the APOE4 gene and fed a diet high in fat and cholesterol exhibit drusen-like structures and retinal atrophy as well as signs of choroidal neovascularization.^38^ The retinal oxidative stress in our mouse model is caused by RPE-specific deletion of *Sod2* using the cre-lox system.^10^ These mice exhibit an age-related decline in the scotopic a-wave and b-wave gradual thinning of the outer nuclear layer. Ultrastructural evidence shows localized atrophy of the retina associated with damage to the underlying RPE and Bruch's membrane. We have used this model to test two drug therapies for retinal atrophy caused by oxidative stress.^13–15^

Despite the fact that gene therapy is in clinical trial for inherited retinal degenerations (Clinicaltrials.gov reference numbers NCT00643747, NCT01482195, NCT02759952, NCT02416622), gene delivery has not been reported in animal models of GA.^39^ Our study demonstrates the development and testing of an antioxidant gene therapy vector in the RPE. This approach results in unambiguous structural and functional protection of rod photoreceptors in a mouse model of progressive retinal degeneration. Increased expression of *Sod2* for a longer time did not have any adverse effect in retinal structure and function. Since RPE-specific expression of *Sod2* rescued both the structure and the function of retina, antioxidant therapy in the RPE protects photoreceptors and bipolar cells, at least indirectly.

At a minimum, gene delivery of *Sod2* to the RPE of mice in which this gene had been deleted revealed that reversion of the genetic lesion could prevent degeneration of the RPE and neural retina in this mouse model. Indeed, protection of photoreceptors and RPE was achieved within 3 months of gene transfer. But we also learned that timing is important: AAV1-*Sod2* injection at 6 weeks, which is prior to the advent of clinical signs of injury in this model, led to substantial protection of the retina based on the ERG and SD-OCT measurements at subsequent times. Subretinal injection of the same vector at 6 months, which is after electrophysiological and structural decline can be documented, did not prevent continued retinal degeneration. We conclude that despite the fact that injury in this model is initiated by mitochondrial oxidative stress, once damage to the RPE is present, antioxidant therapy is not sufficient to protect the retina.

Dietary antioxidant treatment in patients with AMD has shown some benefit. Use of the AREDS formula of antioxidants plus zinc led to a 25% decrease in the number of patients progressing from midstage AMD to neovascular disease.^5^ Nevertheless, the same treatment did not prevent progression of early dry AMD to GA. By analogy to our results, we conclude that once tissue damage and inflammatory processes such as complement dysregulation are under way, antioxidant therapy is not sufficient to prevent death of RPE cells and damage to the choriocapillaris and photoreceptor cells. In contrast, dietary consumption of antioxidant nutrients may reduce the incidence of early AMD in people with high-risk alleles.^40,41^ We conclude that antioxidant gene therapy may prevent the progression of dry AMD if used early in the course of the disease, but such an intervention would require identifying patients with early AMD who are likely to progress to GA, and such prediction may be challenging. The contralateral eye of patients with unilateral choroidal neovascularization or GA would be a likely place to start.

## Supplementary Material

Supplement 1

## References

[1] Chader GJ, Taylor A. Preface: the aging eye: normal changes, age-related diseases, and sight-saving approaches. *Invest Ophthalmol Vis Sci*. 2013; 54: ORSF1–ORSF4. 24335060 10.1167/iovs.13-12993 PMC4139274

[2] Fernández-Robredo P, Sancho A, Johnen S, Current treatment limitations in age-related macular degeneration and future approaches based on cell therapy and tissue engineering. *J Ophthalmol*. 2014; 2014: 510285. 24672707 10.1155/2014/510285 PMC3941782

[3] Schwartz SD, Tan G, Hosseini H, Nagiel A. Subretinal transplantation of embryonic stem cell-derived retinal pigment epithelium for the treatment of macular degeneration: an assessment at 4 years. *Invest Ophthalmol Vis Sci*. 2016; 57: ORSFc1–ORSFc9. 27116660 10.1167/iovs.15-18681

[4] Imamura Y, Noda S, Hashizume K, Drusen, choroidal neovascularization, and retinal pigment epithelium dysfunction in SOD1-deficient mice: a model of age-related macular degeneration. *Proc Natl Acad Sci U S A*. 2006; 103: 11282–11287. 16844785 10.1073/pnas.0602131103 PMC1544079

[5] Age-Related Eye Disease Study Research Group A randomized, placebo-controlled, clinical trial of high-dose supplementation with vitamins C and E, beta carotene, and zinc for age-related macular degeneration and vision loss: AREDS report no. 8. *Arch Ophthalmol*. 2001; 119: 1417–1436. 11594942 10.1001/archopht.119.10.1417 PMC1462955

[6] Hanus J, Zhao F, Wang S. Current therapeutic developments in atrophic age-related macular degeneration. *Br J Ophthalmol*. 2016; 100: 122–127. 26553922 10.1136/bjophthalmol-2015-306972 PMC4944382

[7] Broadhead GK, Grigg JR, Chang AA, McCluskey P. Dietary modification and supplementation for the treatment of age-related macular degeneration. *Nutr Rev*. 2015; 73: 448–462. 26081455 10.1093/nutrit/nuv005

[8] Querques G, Rosenfeld PJ, Cavallero E, Treatment of dry age-related macular degeneration. *Ophthalmic Res*. 2014; 52: 107–115. 25228171 10.1159/000363187

[9] Bowes Rickman C, Farsiu S, Toth CA, Klingeborn M. Dry age-related macular degeneration: mechanisms, therapeutic targets, and imaging. *Invest Ophthalmol Vis Sci*. 2013; 54: ORSF68–ORSF80. 24335072 10.1167/iovs.13-12757 PMC3864379

[10] Mao H, Seo SJ, Biswal MR, Mitochondrial oxidative stress in the retinal pigment epithelium leads to localized retinal degeneration. *Invest Ophthalmol Vis Sci*. 2014; 55: 4613–4627. 24985474 10.1167/iovs.14-14633 PMC4112607

[11] Seo S, Krebs MP, Mao H, Jones K, Conners M, Lewin AS. Pathological consequences of long-term mitochondrial oxidative stress in the mouse retinal pigment epithelium. *Exp Eye Res*. 2012; 101: 60–71. 22687918 10.1016/j.exer.2012.05.013 PMC3419481

[12] Justilien V, Pang J-J, Renganathan K, SOD2 knockdown mouse model of early AMD. *Invest Ophthalmol Vis Sci*. 2007; 48: 4407–4420. 17898259 10.1167/iovs.07-0432 PMC6549721

[13] Ahmed CM, Biswal MR, Li H, Han P, Ildefonso CJ, Lewin AS. Repurposing an orally available drug for the treatment of geographic atrophy. *Mol Vis*. 2016; 22: 294–310. 27110092 PMC4818958

[14] Biswal MR, Ahmed CM, Ildefonso CJ, Systemic treatment with a 5HT1a agonist induces anti-oxidant protection and preserves the retina from mitochondrial oxidative stress. *Exp Eye Res*. 2015; 140: 94–105. 26315784 10.1016/j.exer.2015.07.022 PMC4624518

[15] Thampi P, Rao HV, Mitter SK, The 5HT1a receptor agonist 8-Oh DPAT induces protection from lipofuscin accumulation and oxidative stress in the retinal pigment epithelium. *PLoS One*. 2012; 7: e34468. 22509307 10.1371/journal.pone.0034468 PMC3317995

[16] Berkowitz BA, Lewin AS, Biswal MR, Bredell BX, Davis C, Roberts R. MRI of retinal free radical production with laminar resolution in vivo. *Invest Ophthalmol Vis Sci*. 2016; 57: 577–585. 26886890 10.1167/iovs.15-18972 PMC4771178

[17] Hauswirth WW, Aleman TS, Kaushal S, Treatment of leber congenital amaurosis due to RPE65 mutations by ocular subretinal injection of adeno-associated virus gene vector: short-term results of a phase I trial. *Hum Gene Ther*. 2008; 19: 979–990. 18774912 10.1089/hum.2008.107 PMC2940541

[18] Maguire AM, Simonelli F, Pierce EA, Safety and efficacy of gene transfer for Leber's congenital amaurosis. *N Engl J Med*. 2008; 358: 2240–2248. 18441370 10.1056/NEJMoa0802315 PMC2829748

[19] Cideciyan AV, Aguirre GK, Jacobson SG, Pseudo-fovea formation after gene therapy for RPE65-LCA. *Invest Ophthalmol Vis Sci*. 2014; 56: 526–537. 25537204 10.1167/iovs.14-15895 PMC4303042

[20] MacLaren RE, Bennett J, Schwartz SD. Gene therapy and stem cell transplantation in retinal disease: the new frontier. *Ophthalmology*. 2016; 123: S98–S106. 27664291 10.1016/j.ophtha.2016.06.041 PMC5545086

[21] Le Y-Z, Zheng W, Rao P-C, Inducible expression of cre recombinase in the retinal pigmented epithelium. *Invest Ophthalmol Vis Sci*. 2008; 49: 1248–1253. 18326755 10.1167/iovs.07-1105 PMC2711689

[22] Haire SE, Pang J, Boye SL, Light-driven cone arrestin translocation in cones of postnatal guanylate cyclase-1 knockout mouse retina treated with AAV-GC1. *Invest Ophthalmol Vis Sci*. 2006; 47: 3745–3753. 16936082 10.1167/iovs.06-0086 PMC1761699

[23] Zolotukhin S, Potter M, Zolotukhin I, Production and purification of serotype 1, 2, and 5 recombinant adeno-associated viral vectors. *Methods*. 2002; 28: 158–167. 12413414 10.1016/s1046-2023(02)00220-7

[24] Timmers AM, Zhang H, Squitieri A, Gonzalez-Pola C. Subretinal injections in rodent eyes: effects on electrophysiology and histology of rat retina. *Mol Vis*. 2001; 7: 131–137. 11435999

[25] Auricchio A, Kobinger G, Anand V, Exchange of surface proteins impacts on viral vector cellular specificity and transduction characteristics: the retina as a model. *Hum Mol Genet*. 2001; 10: 3075–3081. 11751689 10.1093/hmg/10.26.3075

[26] Chen B, Caballero S, Seo S, Grant MB, Lewin AS. Delivery of antioxidant enzyme genes to protect against ischemia/reperfusion-induced injury to retinal microvasculature. *Invest Ophthalmol Vis Sci*. 2009; 50: 5587–5595. 19628743 10.1167/iovs.09-3633 PMC3756491

[27] Liu Y, Tang L, Chen B. Effects of antioxidant gene therapy on retinal neurons and oxidative stress in a model of retinal ischemia/reperfusion. *Free Radic Biol Med*. 2012; 52: 909–915. 22240151 10.1016/j.freeradbiomed.2011.12.013

[28] Wu J, Peachey NS, Marmorstein AD. Light-evoked responses of the mouse retinal pigment epithelium. *J Neurophysiol*. 2004; 91: 1134–1142. 14614107 10.1152/jn.00958.2003 PMC2897140

[29] Ferrington DA, Kapphahn RJ, Leary MM, Increased retinal mtDNA damage in the CFH variant associated with age-related macular degeneration. *Exp Eye Res*. 2016; 145: 269–277. 26854823 10.1016/j.exer.2016.01.018 PMC4842097

[30] Ferrington DA, Sinha D, Kaarniranta K. Defects in retinal pigment epithelial cell proteolysis and the pathology associated with age-related macular degeneration. *Prog Retin Eye Res*. 2016; 51: 69–89. 26344735 10.1016/j.preteyeres.2015.09.002 PMC4769684

[31] Gouras P, Ivert L, Neuringer M, Nagasaki T. Mitochondrial elongation in the macular RPE of aging monkeys, evidence of metabolic stress. *Graefes Arch Clin Exp Ophthalmol*. 2016; 254: 1221–1227. 27106622 10.1007/s00417-016-3342-x PMC4884660

[32] Pujol-Lereis LM, Schäfer N, Kuhn LB, Rohrer B, Pauly D. Interrelation between oxidative stress and complement activation in models of age-related macular degeneration. *Adv Exp Med Biol*. 2016; 854: 87–93. 26427398 10.1007/978-3-319-17121-0_13

[33] Gao J, Liu RT, Cao S, NLRP3 inflammasome: activation and regulation in age-related macular degeneration. *Mediators Inflamm*. 2015; 2015: 690243. 25698849 10.1155/2015/690243 PMC4324923

[34] Chang Y-C, Chang W-C, Hung K-H, The generation of induced pluripotent stem cells for macular degeneration as a drug screening platform: identification of curcumin as a protective agent for retinal pigment epithelial cells against oxidative stress. *Front Aging Neurosci*. 2014; 6: 191. 25136316 10.3389/fnagi.2014.00191 PMC4117985

[35] Cano M, Thimmalappula R, Fujihara M, Cigarette smoking, oxidative stress, the anti-oxidant response through Nrf2 signaling, and age-related macular degeneration. *Vision Res*. 2010; 50: 652–664. 19703486 10.1016/j.visres.2009.08.018 PMC3575185

[36] Zhao Z, Chen Y, Wang J, Age-related retinopathy in NRF2-deficient mice. *PLoS One*. 2011; 6: e19456. 21559389 10.1371/journal.pone.0019456 PMC3084871

[37] Hahn P, Qian Y, Dentchev T, Disruption of ceruloplasmin and hephaestin in mice causes retinal iron overload and retinal degeneration with features of age-related macular degeneration. *Proc Natl Acad Sci U S A*. 2004; 101: 13850–13855. 15365174 10.1073/pnas.0405146101 PMC518844

[38] Malek G, Johnson LV, Mace BE, Apolipoprotein E allele-dependent pathogenesis: a model for age-related retinal degeneration. *Proc Natl Acad Sci U S A*. 2005; 102: 11900–11905. 16079201 10.1073/pnas.0503015102 PMC1187976

[39] Catchpole I, Germaschewski V, Hoh Kam J, Systemic administration of Abeta mAb reduces retinal deposition of Abeta and activated complement C3 in age-related macular degeneration mouse model. *PLoS One*. 2013; 8: e65518. 23799019 10.1371/journal.pone.0065518 PMC3682980

[40] Bisti S, Maccarone R, Falsini B. Saffron and retina: neuroprotection and pharmacokinetics. *Vis Neurosci*. 2014; 31: 355–361. 24819927 10.1017/S0952523814000108

[41] Ho L, van Leeuwen R, Witteman JCM, Reducing the genetic risk of age-related macular degeneration with dietary antioxidants, zinc, and ω-3 fatty acids: the Rotterdam study. *Arch Ophthalmol*. 2011; 129: 758–766. 21670343 10.1001/archophthalmol.2011.141

