# Supplementary material for: Timing of Antioxidant Gene Therapy: Implications for Treating Dry AMD
Source: Invest Ophthalmol Vis Sci. 2017 Feb;58(2):1237–45. doi: 10.1167/iovs.16-21272 (PMC5338629; doi:10.1167/iovs.16-21272)
Supplement: Supplement 1 [file iovs-58-02-36_s01.pdf]

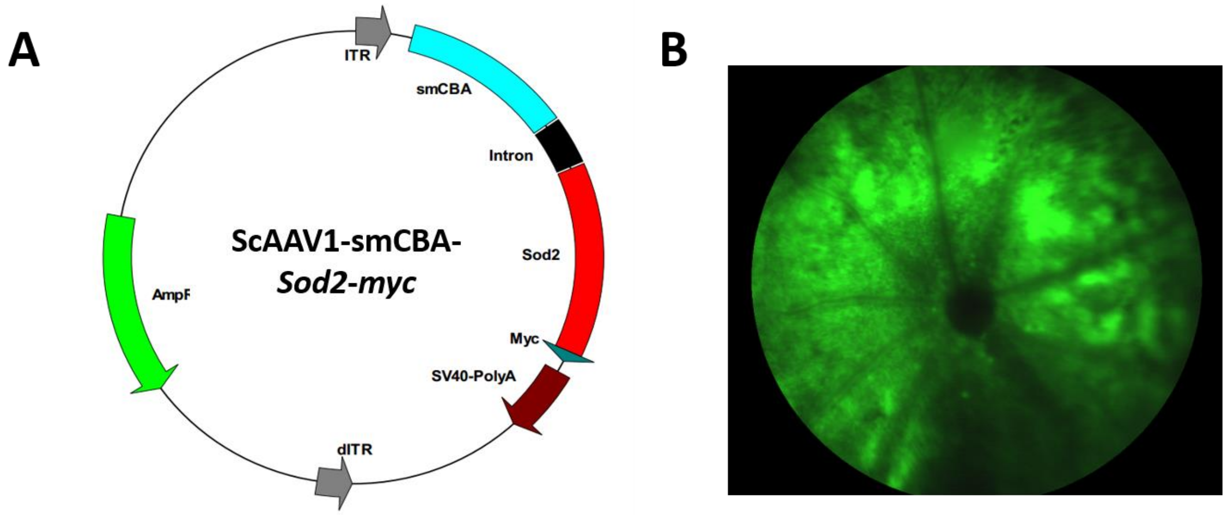

SUPPLEMENTAL FIGURE 1. Vector design. Self-complementary AAV vector was used to express the *Sod2* cDNA with a single copy of myc epitope inserted replacing stop codon of *Sod2* (A). A scAAV vector carrying GFP cDNA used as a control to compare the effects of *Sod2* transgene expression. Fundus image shows wide spread GFP expression in the retina (B) by subretinal delivery of AAV1-GFP vector, validating the injection procedure.

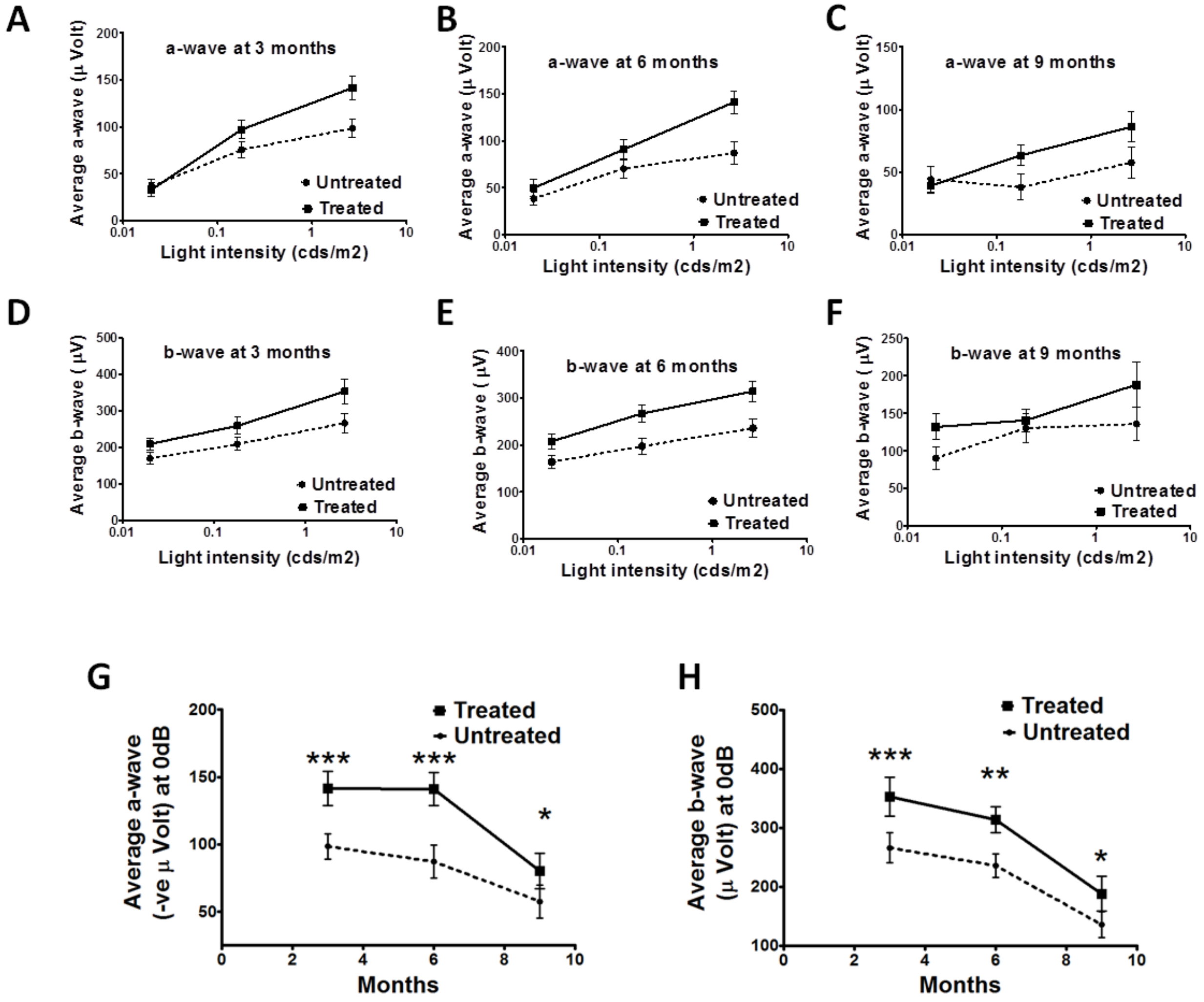

SUPPLEMENTAL FIGURE 2. ERG at different light intensity and time points. A, C, and E show stabilization of a-wave amplitudes 3, 6 and 9 months after early treatment of AAV1-*Sod2* vector (n = 18) compared to control eyes treated with AAV1-*GFP* vector (n =18) at different light intensities. Similarly, B, D, and F shows relative preservation of b-wave amplitudes for the same time points. G and H shows preservation of a and b-wave responses over 9 months following early treatment of AAV1-*Sod2* vector (n = 7) compared to control eyes injected with AAV1-GFP vector (n = 7) at a highest light intensity. *** $P < 0.001$, ** $P < 0.01$, * $P < 0.05$.

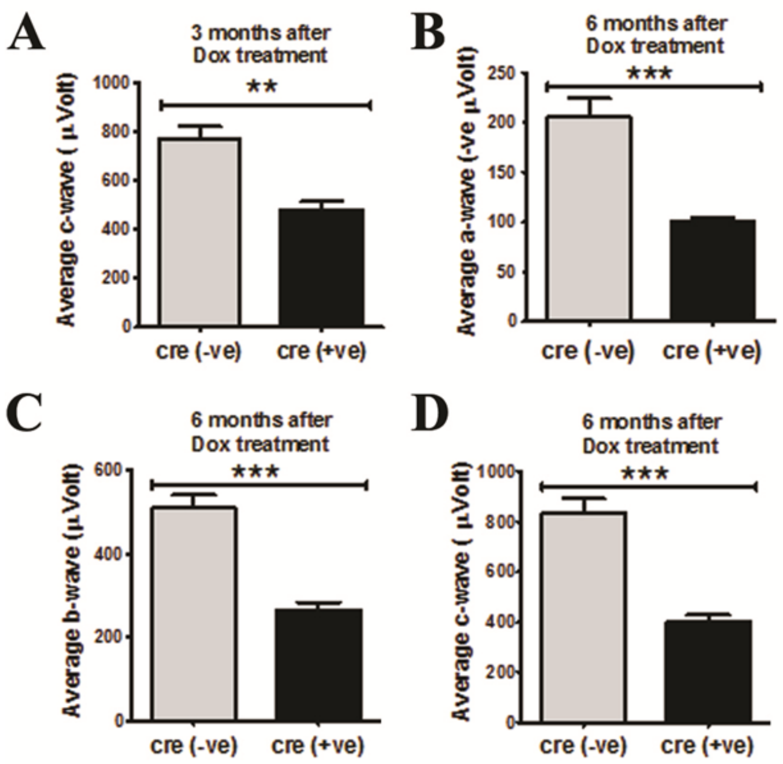

SUPPLEMENTAL FIGURE 3. Relative loss of ERG amplitudes following deletion of Sod2. (A) The average c-wave amplitude was significantly decreased 3 months following doxycycline treatment in *Sod2* $^{flox/flox}$-*VMD2-cre* mice (n = 7, ** P = 0.004) compared to littermate *Sod2* $^{flox/flox}$ mice (n= 9). Compared to *Sod2* $^{flox/flox}$ mice (n = 10), *Sod2* $^{flox/flox}$-*VMD2-cre mice* (n = 14, *** P < 0.0001) showed a significant loss of a-wave (B), b-wave (C) and c-wave (D) 6 months following doxycycline treatment.

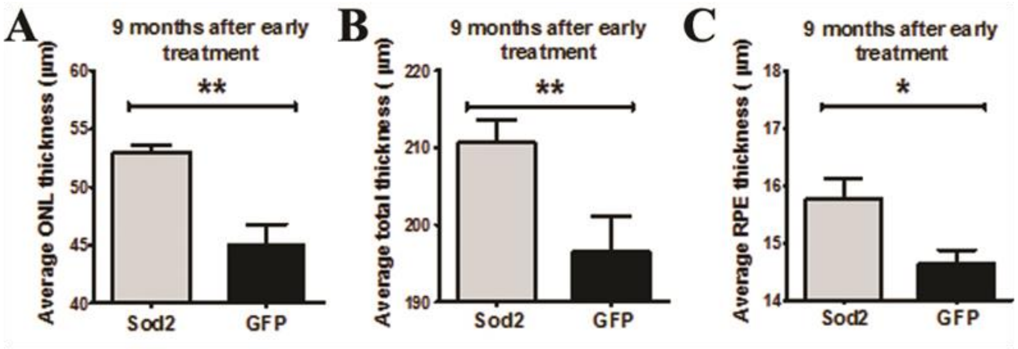

SUPPLEMENTAL FIGURE 4. Retinal thickness by SD-OCT 9 months following early treatment. Thinning of outer nuclear thickness (ONL) was significantly delayed (A) 9 months following early treatment of AAV1-Sod2 vector compared to control eyes injected with AAV1-GFP vector. Upon measuring total thickness and RPE thickness, AAV1-Sod2 injected eyes showed preservation of both total thickness (B) and RPE thickness (C) compared to control eyes injected with AAV1-GFP vector.

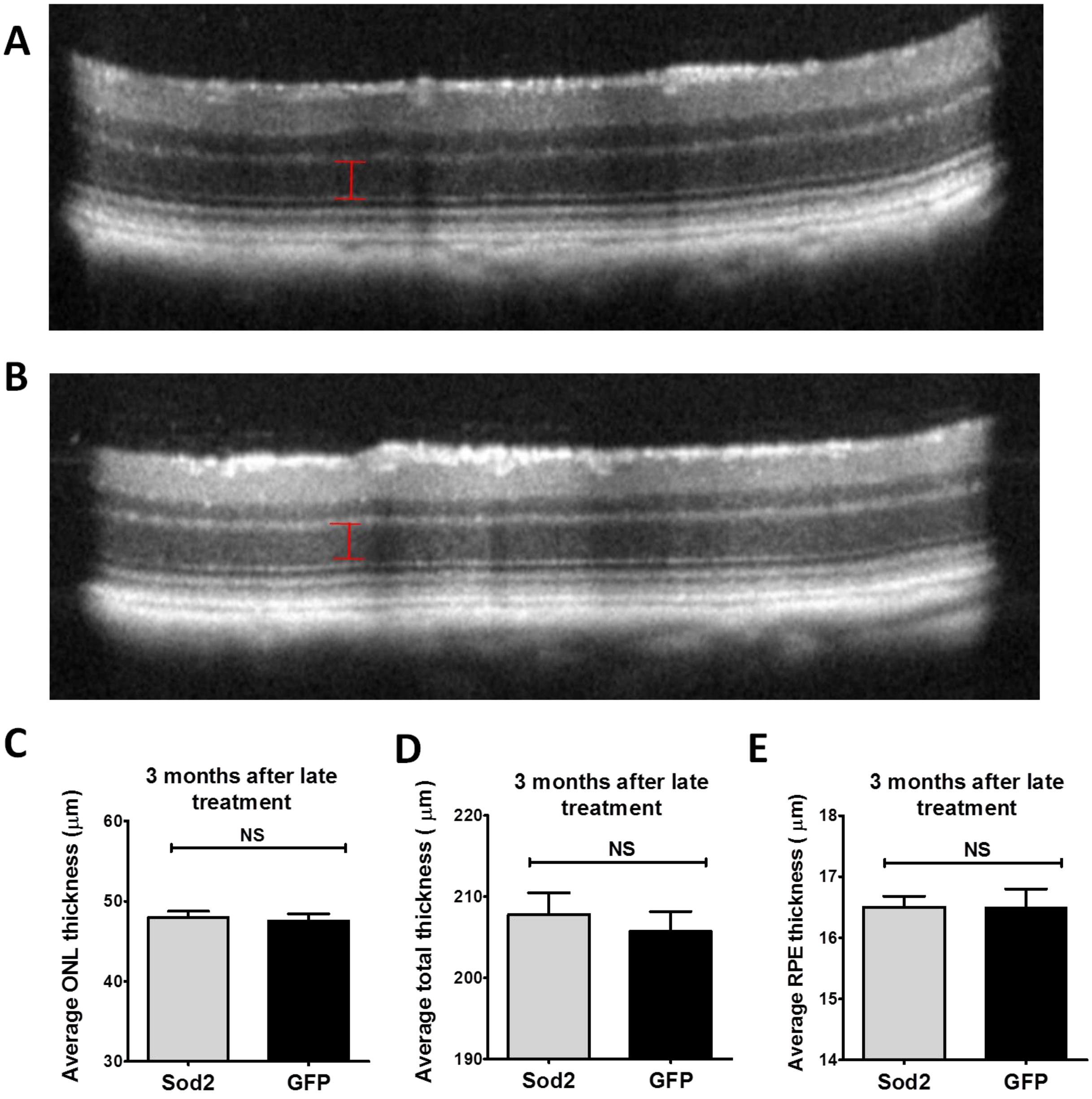

SUPPLEMENTAL FIGURE 5. SD-OCT 3 months after late treatment. SD-OCT images of mouse retinas from mice treated late with AAV1-*Sod2* (A) compared to control treated eyes treated with AAV1-GFP (B). We did not detect any difference in retinal dimensions: ONL thickness (C), Total thickness (D), and RPE Thickness (E) between AAV1-*Sod2* treated and control treated eyes injected at 6 months of age.
